# Supplementary material for: Defining an intermediate category of tuberculin skin test: A mixture model analysis of two high-risk populations from Kampala, Uganda
Source: PLoS One. 2021 Jan 22;16(1):e0245328. doi: 10.1371/journal.pone.0245328 (PMC7822548; doi:10.1371/journal.pone.0245328)
Supplement: S1 File — (DOCX) [file pone.0245328.s001.docx]

**S1 File. Data collection of participant’s characteristics (English and Luganda versions).**
