# Supplementary material for: Defining an intermediate category of tuberculin skin test: A mixture model analysis of two high-risk populations from Kampala, Uganda
Source: PLoS One. 2021 Jan 22;16(1):e0245328. doi: 10.1371/journal.pone.0245328 (PMC7822548; doi:10.1371/journal.pone.0245328)
Supplement: Attachment — Submitted filename: ester,s review comments.docx [file pone.0245328.s002.docx]

**Reviewer’s comments**

In this study entiled: Defining an indeterminate tuberculin skin test: a mixture model analysis of two high-risk populations from Kampala, Uganda
This is a well-intended initiative to address a major problem affecting the African population.

1The study, context is in keeping with other literature on the topic. Latent TB remains a pool for active TB disease and treatment of latent TB would be a better elimination strategy.

2 Indeed the readily available TST is not well interpreted because of cross reaction of the BCG vaccine and non tuberculosis bacteria. Consequently the identification of the true latent infected individuals may sometimes be missed out. The other tests like IGRA for latent TB diagnosis is not validated endemic areas. Therefore the most popular test may remain TST.

3 The	authors therefore set out to map out the epidemiological distribution of TST in two study populations of urban Kampala using mixture model method and to define criteria for diagnosis of the TST test.

4 The selected study populations were appropriate, Kawempe division one of the crowding areas of Kampala yet crowding is among the well known risk factors for mtb transmission.

5 Sample size and study duration for both studies were adequate to generate sufficient data for the analysis.

6 The mixture model method used was appropriate since they were dealing with intractable variables and data was from two different populations.

The Lubaga division included controls in their study. The Kawempe study however did not include controls.

7Hartigans’ dip test of unimodality to asses distribution was unimodal or multimodal was appropriate .

8 The Finite mixture normal model was for heterogeneity was appropriate.

9 presentations of data available is appropriate with tables and figures supporting it

10The authors were able to determine an intermediate reading they referred to as indeterminate.

**Reviewer’s comments**

In view of the above observations, the this study, Defining an indeterminate tuberculin skin test: a mixture model analysis of two high-risk populations from Kampala, Uganda

I am only concerned about the Kawempe study that did not have a control group.

Are these findings generalized to other populations at risk of disease?

On the whole I find this study appropriate for publication.
